# Pocket2Drug: An Encoder-Decoder Deep Neural Network for the Target-Based Drug Design

**DOI:** 10.3389/fphar.2022.837715

**Published:** 2022-03-11

**Authors:** Wentao Shi, Manali Singha, Gopal Srivastava, Limeng Pu, J. Ramanujam, Michal Brylinski

**Affiliations:** ^1^ Division of Electrical and Computer Engineering, Louisiana State University, Baton Rouge, LA, United States; ^2^ Department of Biological Sciences, Louisiana State University, Baton Rouge, LA, United States; ^3^ Center for Computation and Technology, Louisiana State University, Baton Rouge, LA, United States

**Keywords:** ligand binding sites, drug discovery and development, in silico drug design, deep learning, graph neural network, recurrent neural network, generative model, machine learning

## Abstract

Computational modeling is an essential component of modern drug discovery. One of its most important applications is to select promising drug candidates for pharmacologically relevant target proteins. Because of continuing advances in structural biology, putative binding sites for small organic molecules are being discovered in numerous proteins linked to various diseases. These valuable data offer new opportunities to build efficient computational models predicting binding molecules for target sites through the application of data mining and machine learning. In particular, deep neural networks are powerful techniques capable of learning from complex data in order to make informed drug binding predictions. In this communication, we describe Pocket2Drug, a deep graph neural network model to predict binding molecules for a given a ligand binding site. This approach first learns the conditional probability distribution of small molecules from a large dataset of pocket structures with supervised training, followed by the sampling of drug candidates from the trained model. Comprehensive benchmarking simulations show that using Pocket2Drug significantly improves the chances of finding molecules binding to target pockets compared to traditional drug selection procedures. Specifically, known binders are generated for as many as 80.5% of targets present in the testing set consisting of dissimilar data from that used to train the deep graph neural network model. Overall, Pocket2Drug is a promising computational approach to inform the discovery of novel biopharmaceuticals.

## Introduction

Recent developments in genomics revealed novel disease-related molecular targets, many of which are yet to be characterized with respect to the possibility of modulating their functions with pharmaceutical agents. Another challenge in pharmacotherapy arises from resistance effects to existing drugs complicating the treatment of particularly infectious diseases (Trebosc et al., 2019) and cancer (Shou et al., 2004). Therefore, many drug development projects are focused on the discovery of small molecule therapeutics with new mode of action (Gerry and Schreiber, 2018). Generating novel small molecules is a difficult endeavor due to the high complexity of biological systems and the enormous size of chemical space of organic compounds. Traditional experimental techniques can be used to identify drug-like molecules performing specific biochemical tasks by binding to macromolecular targets with a high specificity in order to modulate their cellular functions. Nonetheless, even advanced high-throughput screening methods have notable limitations due to the long time and high costs of screening a large number of drug candidates.

To make the drug discovery process more efficient, modern approaches incorporate miscellaneous computational components. Virtual screening (VS) is perhaps the most widely used strategy to help identify potentially bioactive molecules from large collections of commercially available as well as virtual compounds (Segler et al., 2018). Despite its utility, this technology has certain drawbacks such as high false-positive rates, the requirement of predefined ligand libraries for structure-based VS, oversimplified scoring functions, and protein structure frameworks absent in ligand-based VS (Wu et al., 2019). More recently, machine learning (ML) methods addressing many of these issues have become available for drug discovery. New ML techniques include a quantitative structure-activity relationship model to predict the target affinity, toxicity, and side effects (Mouchlis et al., 2021) and an approach to model polypharmacy side effects with graph convolutional networks (GCN) (Zitnik et al., 2018).

Deep learning (DL) is a family of modern machine leaning models utilizing deep neural networks (DNNs). DL models have been demonstrated to be powerful feature extractors for ligand binding site classifiers (Jiménez et al., 2017; Pu et al., 2019; Shi et al., 2020) and metric learning models for binding sites in proteins (Simonovsky and Meyers, 2020). Recurrent neural networks (RNNs) are iterative DL models that generate sequences through multiple iterations. In each iteration, the RNN model generates an output of time t taking the output of iteration t−1 as the input. According to the probabilistic language model (Graves, 2013), the probability of input token $x_{t+1}$ is modeled as $P\left(x_{t+1}|y_{t}\right)$ , which is the probability of $x_{t+1}$ conditioned on the output token $y_{t}$ from the previous iteration. This powerful methodology was applied to *de novo* drug discovery, where RNNs were trained to model the probability distribution of a drug dataset (Ertl et al., 2017; Segler et al., 2018; Gupta et al., 2018; Yasonik, 2020). These methods treat a drug dataset as a set of languages and employ an RNN to learn the corresponding language models. After the training stage is completed, the RNN learns the probability distribution P(molecule) of the drug dataset, from which molecules can be sampled. RNN-based approaches often represent molecules using a simplified molecular-input line-entry system (SMILES) (Weininger, 1988), where individual string characters represent tokens of time steps. Although using RNNs to learn the distributions of drug datasets offers new opportunities to find drugs, these techniques still employ a random search of the chemical space leading to long virtual screening times. From a computational standpoint, when the aim is to identify promising lead molecules against a target binding site, it is certainly advantageous to have the search space significantly reduced.

In order to achieve this goal, we developed Pocket2Drug, a new deep generative model with the encoder-decoder architecture. Inspired by the framework of image captioning models taking images as the input to generate corresponding captions (Vinyals et al., 2015; Xu et al., 2015), the basic idea is to provide RNN with the prior information on ligand binding pockets to improve the chances of finding bioactive molecules. A typical image captioning model consists of two parts, an encoder/feature extractor and a decoder. A convolutional neural network (CNN) is often used as the encoder extracting fixed-size latent feature vectors from the input images containing the prior information that can subsequently be decoded by an RNN to generate image captions. Formally, image captioning models learn the probability of sequences conditioned on prior information, i.e., P(caption|image) .

Pocket2Drug has a similar encoder-decoder architecture consisting of an encoder to extract features and a decoder to generate molecules. Nonetheless, Pocket2Drug differs from typical image captioning models in that it employs a graph representation of drug binding sites instead of images. Consequently, a GNN is employed as the encoder to extract the prior information from input pockets followed by an RNN decoder to generate molecule strings, which are the equivalents of image captions. In comprehensive benchmarking simulations against ligand-bound, ligand-free, and low-homology datasets of binding sites, we show that Pocket2Drug employing the encoder-decoder DNN effectively predicts binding drugs for input pocket structures.

## Materials and Methods

### Datasets

Datasets used in this study were compiled from a non-redundant library of 51,677 pockets with bound ligands constructed for binding site prediction with *e*FindSite (Brylinski and Feinstein, 2013). The redundancy in the original library was already removed by excluding proteins with the template modeling (TM)-score, measuring the structure similarity (Zhang and Skolnick, 2004), of ≥0.4 and the 3D Tanimoto coefficient (TC), measuring the ligand similarity (Kawabata, 2011), of ≥0.7. We further filtered the dataset based on the synthetic accessibility (SA) score (Ertl and Schuffenhauer, 2009) removing low- and high-complexity compounds whose SA scores are ≤1 and ≥6, respectively. This procedure resulted in a high-quality dataset of 48,365 pockets binding small organic compounds, which were randomly split into training (90%) and testing (10%) subsets. The training subset of 43,529 pockets is referred to as the Pocket2Drug-train dataset while the remaining 4,836 (testing) pockets are called the Pocket2Drug-holo dataset.

Next, 433 pockets having a protein sequence identity of ≤0.5 with pockets in the training subset were selected from the Pocket2Drug-holo dataset creating the Pocket2Drug-lowhomol dataset to evaluate the ability to generalize to unseen data. Finally, the basic local alignment search tool (BLAST) (Altschul et al., 1990) was used with a sequence identity threshold of 95% to identify the apo structures of Pocket2Drug-holo proteins in the Protein Data Bank (PDB) (Berman et al., 2002). Ligand-free structures were then aligned on the corresponding holo-proteins with TM-align (Zhang and Skolnick, 2005) and those producing significant alignments with a TM-score of ≥0.5 (Xu and Zhang, 2010) were retained. This procedure resulted in 828 ligand-free pockets referred to as the Pocket2Drug-apo dataset.

### Graph Representation of Pockets

Binding pockets are represented as graphs, in which nodes are non-hydrogen atoms and edges connect pairs of atoms spatially located within 4.5 Å from one another (Shi et al., 2021). Node features include the hydrophobicity (Mahn et al., 2009), the charge, the binding probability (Jian et al., 2016), the solvent accessible surface area (Ali et al., 2014), and the sequence entropy (Liao et al., 2005), whereas the edge attribute is the bond multiplicity for covalently bonded atoms and 0 for atoms interacting non-covalently. Pockets are centered at the origin with principal axes aligned to Cartesian axes. The coordinates of individual atoms are also used as node features in order to provide the additional 3D information on binding pockets. This graph representation of ligand binding sites was used to accurately classify pockets in protein structures with GraphSite (Shi et al., 2021).

### Encoder-Decoder Architecture

**FIGURE 1:**
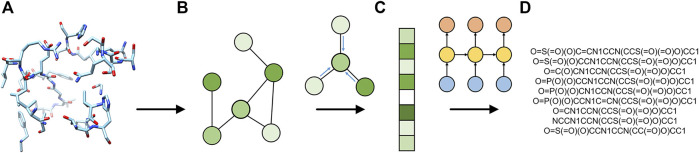
Flowchart of Pocket2Drug. The input ligand-binding pocket **(A)** is first represented as a graph **(B)** and then used by the encoder graph neural network to generate a fixed-size graph embedding **(C)**. The decoder recurrent neural network generates molecule strings **(D)** from the graph embedding.

Pocket2Drug is implemented in PyTorch v1.7.1 (Paszke et al., 2019) and employs a DNN with the encoder-decoder architecture. The model learns the probability distribution of molecules conditioned on ligand binding pockets, P(molecue|pocket) , which is then used to sample molecules for a given pocket as the prior condition. The pipeline implemented in Pocket2Drug is illustrated in Figure 1. For the input binding site (Figure 1A), a graph representation is generated by GraphSite (Shi et al., 2021) (Figure 1B) and the resulting graph is processed by an encoder to generate a fixed-size graph embedding (Figure 1C). As the encoder, we use a GNN constructed by removing the fully connected layers of the GraphSite classifier with parameters pretrained on binding site classification tasks (Shi et al., 2021). Subsequently, an RNN decoder takes the generated embedding vector as the input to compute SMILES sequences representing binding drugs (Figure 1D). Pocket2Drug is trained in an end-to-end fashion meaning that the parameters of both encoder and decoder are updated during backpropagation.

### Graph Neural Network Encoder

The GNN encoder extracts latent features from the input pocket graphs. We use the embedding network implemented in the GraphSite classifier as the feature extractor with the last fully connected layer removed and the remaining parts of the classifier employed as the feature extractor. The message passing function utilizes weighted neighbor node features, in which weights are generated by a two-layer, fully connected neural network taking edge features as the input. Updated node features in k -th layer of node $x_{i}^{\left(k\right)}$ , defined as

$$x_{i}^{\left(k\right)}=h_{\theta }\left(\underset{c\in Channels}{concat}\left(\left(1+\varepsilon_{c}\right)\cdot x_{i}^{\left(k-1\right)}+\sum_{j\in N\left(i\right)}h_{\omega c}\left(e_{ij}\right)\cdot x_{j}^{\left(k-1\right)}\right)\right)$$ (1)

are first computed as a weighted sum of the first-order neighbors. The features of $x_{i}^{\left(k-1\right)}$ are weighted by $\left(1+\epsilon_{c}\right)$ , where $\epsilon_{c}$ is a trainable parameter. The weights of the first-order neighbors are generated by a neural network $h_{\omega c}$ taking the edge feature, $e_{ij}$ , as the input. Then, multiple channels of the weighted sum of the node features are concatenated and updated by another neural network $h_{\theta }$ . Finally, the output of each layer is connected by the jumping knowledge (JK)-network (Xu et al., 2018). The JK-network enables an automatic selection of the number of layers for individual nodes. Finally, the initial node embeddings are processed by the Set2Set graph read-out layer (Vinyals et al., 2016) to construct final, fixed-size graph embeddings.

### Recurrent Neural Network Decoder

**FIGURE 2:**
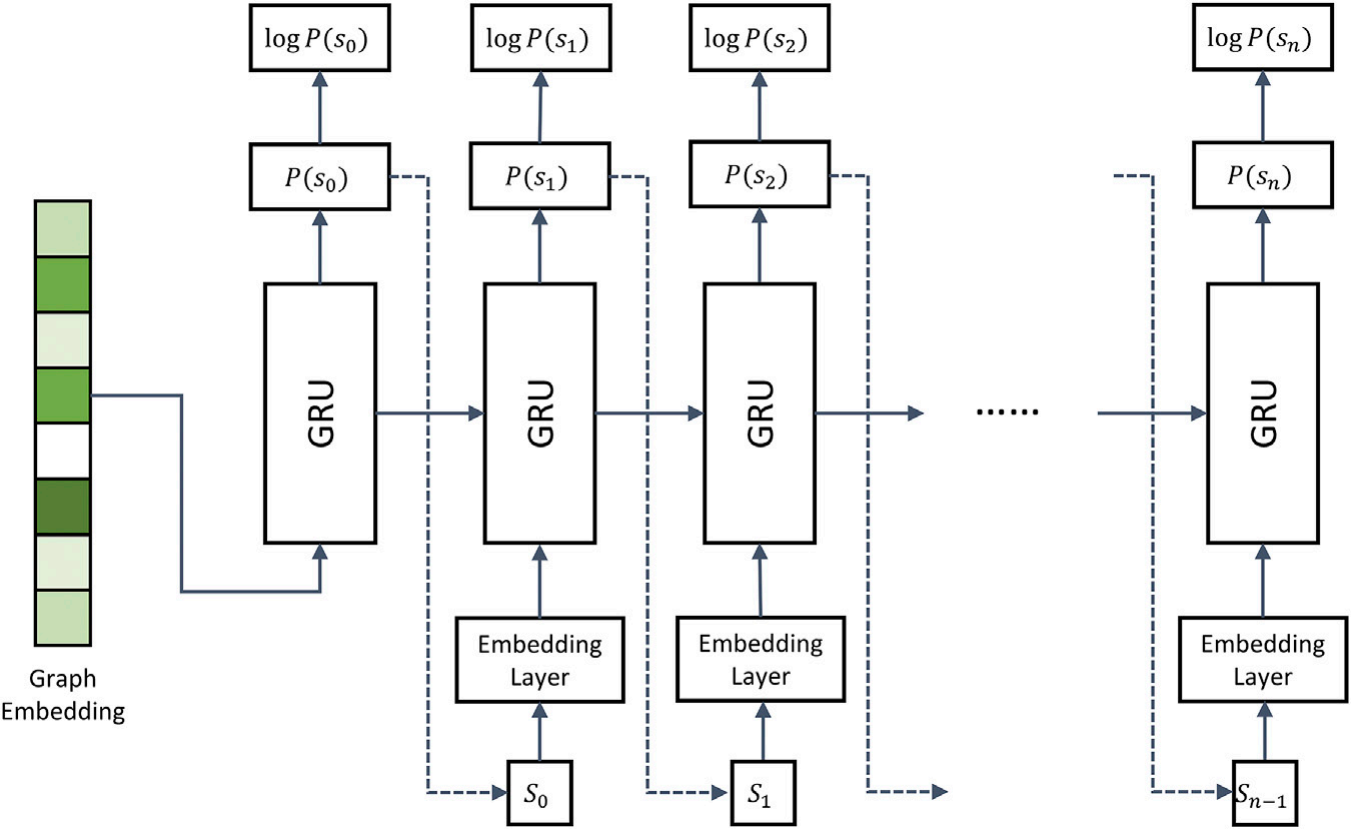
Architecture of the recurrent neural network decoder. The decoder employs multiple gated recurrent units (GRUs). During model training, the molecule strings of binding drugs are used as the input. Dashed arrows represent the inference stage, in which the token sampled from $P\left(s_{t-1}\right)$ is used as the input at iteration t .

As a decoder, we use the gated recurrent unit (GRU), which is a variation of the vanilla RNN (Cho et al., 2014). The decoder network models a conditional probability of the output sequence based on the prior information on a ligand binding pocket:

$$P\left(molecule|pocket\right)=P\left(s_{0}|pocket\right)\prod_{t=1}^{n}P\left(s_{t}|pocket,s_{0},\cdots ,s_{t-1}\right)$$ (2)

where $s_{t}$ is the token of a molecule string at iteration t , and n is the length of the output string. Note that $s_{n}$ represents the “end of string”, or eos , token. Figure 2 shows that the GRU network works differently during training and inference stages. During training, the graph embedding is taken by the GRU as the prior information to model the probability distribution of all tokens, where the probability of a token $s_{0}$ is $P\left(s_{0}\right)$ . In the remaining iterations, input tokens $s_{t}$ of the binding drug string are mapped to vectors by the embedding layer and passed to the GRU as the input. The GRU then predicts the next token by generating another probability distribution $P\left(s_{t+1}\right)$ . The negative log likelihood of the binding drug is used as the loss function:

$$L=-\sum_{t=0}^{n}\log P\left(s_{t}\right)$$ (3)

Dashed arrows in Figure 2 represent the inference stage. Here, the first iteration is the same as during training, i.e., the encoder generates graph embeddings used as the input in the first iteration. However, in the subsequent iterations, the RNN model takes the token $s_{t}$ , sampled from the distribution of the previous step, to generate the distribution $s_{t+1}$ . The inference stops when the eos token is reached.

### Tokenization Scheme

Molecules can be represented by strings encoded by different tokenization schemes. Although SMILES is a widely used molecular string system, it was not designed for ML applications. Because of a strict syntax of SMILES, a significant portion of molecules generated by machine learning models are invalid. In addition, parentheses and ring indicators may be separated by long distances in SMILES strings causing problems for RNNs that have difficulty learning long-term dependencies (Öztürk et al., 2020). This issue can be addressed by improving either the RNN model or the tokenization scheme. For instance, RNN variants implementing “shortcuts” were developed to model long-term dependencies (Hochreiter and Schmidhuber, 1997). A long short-term memory (LSTM) model can also be used instead of a vanilla RNN in *de novo* drug design applications to learn the distribution of a drug dataset (Ertl et al., 2017). Another workaround is to improve the tokenization scheme to make the string representation of molecules more suitable for ML applications. An example is DeepSMILES developed to enhance DL-based models taking SMILES as the input (O’Boyle and Dalke, 2018).

Pocket2Drug employs SELF-referencing Embedding Strings (SELFIES), another molecule tokenization scheme designed for machine learning applications (Krenn et al., 2020). The SELFIES method was selected because of several important properties. Not only any molecule can be represented by a SELFIES string, but also all virtual molecules generated by an ML model are valid. Importantly, the information on rings and branches in SELFIES is localized by storing the branch size and ring size together with their identifiers. This tokenization scheme makes it easier for RNNs to learn from the “past” information compared to, e.g., SMILES that require RNNs to infer ring/branch indicators based on non-localized information.

## Evaluation and Results

**FIGURE 3:**
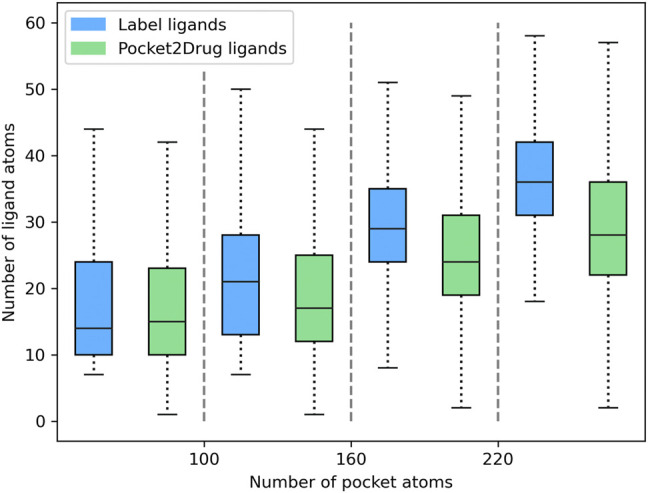
Relationship between the ligand size and the size of binding pockets. The size of ligands and pockets is quantified by the number of non-hydrogen atoms. Binding pockets are assigned to four size groups: <100, 100–160, 161–220, and >220 atoms. For each pocket group, quartiles and the interquartile range are calculated for the size of label ligands (blue bars) and those molecules generated by Pocket2Drug (green bars).

Pocket2Drug was trained on the Pocket2Drug-train dataset and validated against Pocket2Drug-holo, -apo, and -lowhomol datasets. We first analyze the size of molecules generated for the Pocket2Drug-holo dataset. Figure 3 shows that there is a notable correlation between the size of pockets and the size of binding molecules, referred to as label ligands, across experimental complex structures (blue bars). Encouragingly, the size of ligands constructed by Pocket2Drug is also correlated with the pocket size, although these molecules tend to be somewhat smaller than the corresponding label ligands (green bars). This result can be attributed to the fact that capturing longer dependencies in molecular strings is more difficult for the RNN trained to minimize the sum of cross-entropy loss function. In other words, the model makes fewer mistakes by generating smaller molecules.

Next, the quality of molecules generated for the Pocket2Drug-holo dataset is evaluated using two complementing protocols, one based on the chemical similarity of binding molecules (Baldi and Nasr, 2010) and another utilizing the structure alignments of protein pockets (Yeturu and Chandra, 2011). Pocket2Drug is compared to two baselines. The first method randomly selects drug candidates from the ZINC database, a curated collection of commercially available chemical compounds prepared specifically for virtual screening (Irwin and Shoichet, 2005). The second baseline method selects drug candidates from the output of a vanilla RNN (Segler et al., 2018) representing a typical DL-based approach for *de novo* drug design.

### Evaluation by Ligand Chemical Similarity

The performance of Pocket2Drug, ZINC, and vanilla RNN are evaluated with the TC between the generated molecules and label ligands. For each pocket in the Pocket2Drug-holo dataset, TC values are calculated for a specified number of molecules sampled from the model output and the highest TC is selected as the final score. Table 1 reports the percentage of Pocket2Drug-holo pockets with the corresponding score greater than or equal to a TC threshold ranging from 0.7 to 1.0. Encouragingly, using Pocket2Drug significantly improves chances to find binding molecules compared to ZINC and vanilla RNN. For a sample size of 20,480 (10 batches of 2,048 molecules each to maximize the GPU utilization), Pocket2Drug generates at least one molecule which a TC of ≥0.7 to the label ligand for as many as 95.9% pockets. Note that two molecules sharing chemical similarity with a TC of ≥0.7 tend to have a similar bioactivity (Kumar, 2011; Ben Lo, 2016). For the majority of pockets (52.5%), Pocket2Drug selects the label ligand itself (a TC of 1.0). This performance is significantly higher than that of ZINC/vanilla RNN that selects ligands with a TC of ≥0.7 for 58.9%/57.1% of pockets and label ligands for merely 0.4%/0.1% of pockets. Increasing the sample size to 81,920 slightly improves the performance because four times more molecules are used to select that with the highest TC value. A significantly improved performance of Pocket2Drug over vanilla RNN can be attributed to the effective utilization of the prior information on ligand binding pockets learned by the ML model.

**TABLE 1:** Hit rates for the Pocket2Drug-holo dataset.

Method	Sample size of 20,480				Sample size of 81,920			
	*TC ≥ 0.7* (%)	*TC ≥ 0.8* (%)	*TC ≥ 0.9* (%)	*TC =1.0* (%)	*TC ≥ 0.7* (%)	*TC ≥ 0.8* (%)	*TC ≥ 0.9* (%)	*TC =1.0* (%)
Pocket2Drug	95.9	79.9	64.8	52.5	98.4	86.8	69.7	56.4
ZINC	58.9	23.8	3.3	0.4	73.6	40.5	8.4	1.2
Vanilla RNN	57.1	19.7	1.6	0.1	70.9	35.3	4.7	0.3

Next, the performance of Pocket2Drug is assessed against the Pocket2Drug-apo dataset. The mean root-mean-square deviation (RMSD) (Kabsch, 1976) of ligand-free structures against ligand-bound conformations is 1.2 Å ± 0.9. This low RMSD is expected because, with a few exceptions, the structures of apo- and holo-proteins tend to be highly similar (Brylinski and Skolnick, 2008). Table 2 reports hit rates for molecules generated by Pocket2Drug using ligand-free and the corresponding ligand-bound pockets in the Pocket2Drug-holo dataset. Encouragingly, the performance of Pocket2Drug is independent on the ligand binding state of target proteins, therefore, the model does not require input proteins to be co-crystallized with ligands in order to successfully generate binding molecules.

**TABLE 2:** Hit rates for the Pocket2Drug-apo dataset. For each ligand-free structure, the corresponding ligand-bound pocket is selected from the Pocket2Drug-holo dataset for the apples-to-apples comparison.

Binding state	Sample size of 20,480				Sample size of 81,920			
	*TC ≥ 0.7* (%)	*TC ≥ 0.8* (%)	*TC ≥ 0.9* (%)	*TC =1.0* (%)	*TC ≥ 0.7* (%)	*TC ≥ 0.8* (%)	*TC ≥ 0.9* (%)	*TC =1.0* (%)
Ligand-free	95.3	72.7	53.3	37.4	98.2	82.2	57.2	40.5
Ligand-bound	95.3	72.2	52.3	37.0	98.2	81.6	58.1	41.2

We also evaluate the ability of Pocket2Drug to generalize to unseen data by measuring its performance against the Pocket2Drug-lowhomol dataset. As reported in Table 3, label ligands (a TC of 1.0) are generated by Pocket2Drug in 77.1%/80.5% of the cases when the sample size is 20,480/81,920. This performance represents a notable improvement over ZINC and vanilla RNN selecting a very few label ligands. Pocket2Drug also achieves the highest performance for other TC thresholds ranging from 0.7 to 0.9. These results show that Pocket2Drug not only performs exceptionally well against Pocket2Drug-holo and -apo datasets, but also against the Pocket2Drug-lowhomol dataset comprising proteins with a low sequence homology to the training subset demonstrating that it generalizes well to unseen data.

**TABLE 3:** Hit rates for the Pocket2Drug-lowhomol dataset.

Method	Sample size of 20,480				Sample size of 81,920			
	*TC ≥ 0.7* (%)	*TC ≥ 0.8* (%)	*TC ≥ 0.9* (%)	*TC =1.0* (%)	*TC ≥ 0.7* (%)	*TC ≥ 0.8* (%)	*TC ≥ 0.9* (%)	*TC =1.0* (%)
Pocket2Drug	98.2	95.2	87.5	77.1	98.9	96.8	90.0	80.5
ZINC	49.2	18.4	2.7	0.2	66.7	36.3	10.4	2.3
Vanilla RNN	50.8	16.1	0.9	0.0	62.8	28.8	5.7	0.9

**FIGURE 4:**
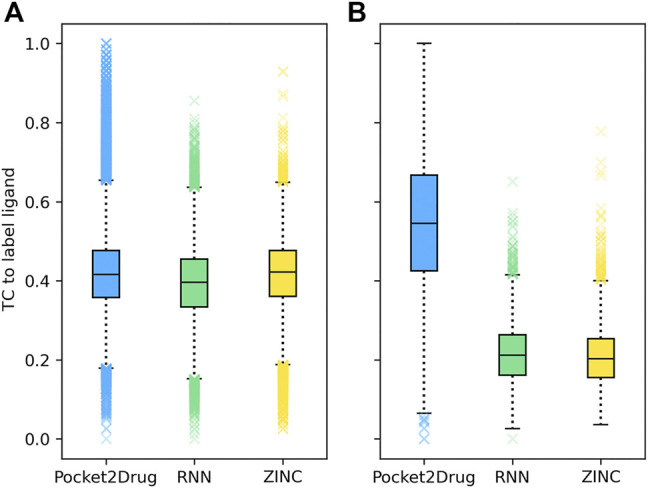
Chemical similarity of molecules generated by Pocket2Drug to label ligands. Label ligands are molecules bound to target pockets in experimental complex structures, **(A)** AMP-PNP binding to MSK1 and **(B)** *ß*-d-allose binding to ALBP. Chemical similarity is measured with the Tanimoto coefficient (TC).

Two representative examples of pockets in the Pocket2Drug-lowhomol dataset are discussed in detail, a nucleotide binding site in the human mitogen and stress activated protein kinase 1 (MSK1) and a sugar binding site in d-allose binding protein (ALBP) from *E*. *coli*. MSK1 is involved in the regulation of mitogen activated kinases and it is required by the tumor-promoter-induced neoplastic cell transformation (Malakhova et al., 2010). The complex structure of MSK1 and the phospho-amino-phosphonic acid-adenylate ester (AMP-PNP) (Malakhova et al., 2010) was chosen as the target. AMP-PNP is a competitive ATPase inhibitor blocking the ATP-dependent oxidative phosphorylation (Lardy et al., 1975). Figure 4A shows the distribution of TC similarities between the label ligand, AMP-PNP, and molecules generated by Pocket2Drug and two baseline methods. Although most virtual molecules have relatively low TC similarities to AMP-PNP, more molecules with high TC vales are sampled from the Pocket2Drug model compared to ZINC and vanilla RNN. According to the Fisher-Pitman permutation test (Neuhäuser and Manly, 2004), the difference between Pocket2Drug and vanilla RNN is statistically significant with a *p*-value close to 0 and that between Pocket2Drug and ZINC is insignificant with a *p*-value of 0.1.

**FIGURE 5:**
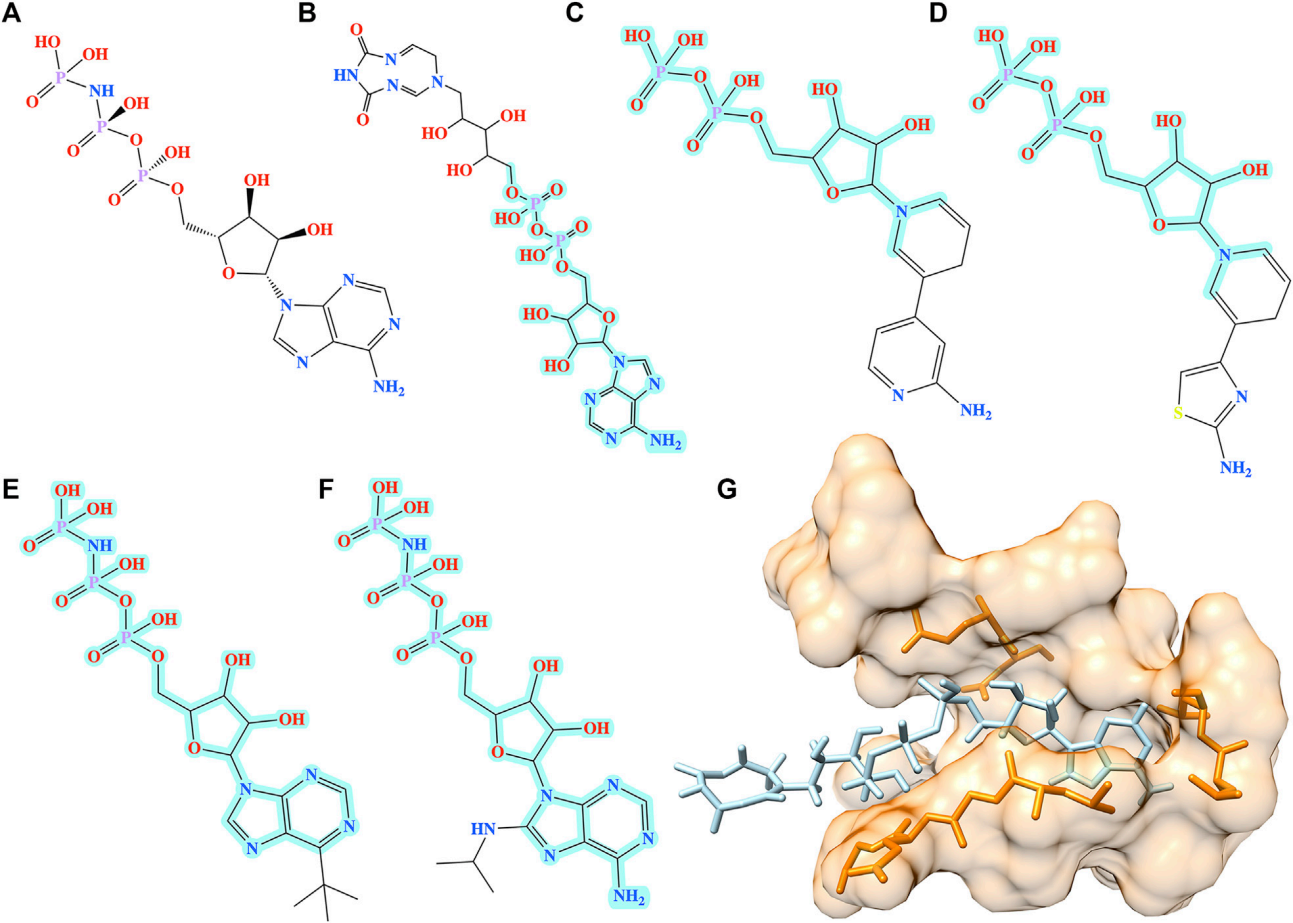
Examples of molecules generated by Pocket2Drug for a binding site in MSK1. **(A)** The label ligand, AMP-PNP. **(B–F)** Molecules constructed by Pocket2Drug with maximum common substructures to the label ligand highlighted in cyan. **(G)** Molecule shown in **B** (ice blue) docked to the binding site in MSK1 (orange).

To better understand the biological relevance of molecules generated by Pocket2Drug, five representative compounds with TC similarities against AMP-PNP ranging from 1.0 to 0.8 are presented in Figure 5. Figure 5A shows AMP-PNP, which is a nonhydrolyzable ATP analogue forming hydrogen bonds with MSK1 pocket residues through several moieties, NH_2_ in adenine, 3′ OH in pentose sugar, OH in *ß*-phosphate, NH linking *ß*- and γ-phosphates and OH in γ-phosphate in the complex crystal structure (Malakhova et al., 2010). Interestingly, several molecules generated by Pocket2Drug have common substructures with either substitutions in the adenine moiety (Figures 5E,F) and the terminal phosphate group (Figure 5B) or sharing the PNP subunit (Figures 5C,D). These virtual molecules contain groups forming important hydrogen bonds with MSK1 pocket residues. To further evaluate the possibility of binding, all molecules were docked into the AMP-PNP pocket of MSK1 with fkcombu (Kawabata and Nakamura, 2014). The docking scores of the generated molecules are 12.5, 18.1, 21.8, 17.6, and 13.0 (Figures 5B–F, respectively). These results indicate that molecules generated by Pocket2Drug dock favorably to the target pocket with the compound shown in Figures 5B,G having the best docking score due to the substitution in *ß*-phosphate group.

**FIGURE 6:**
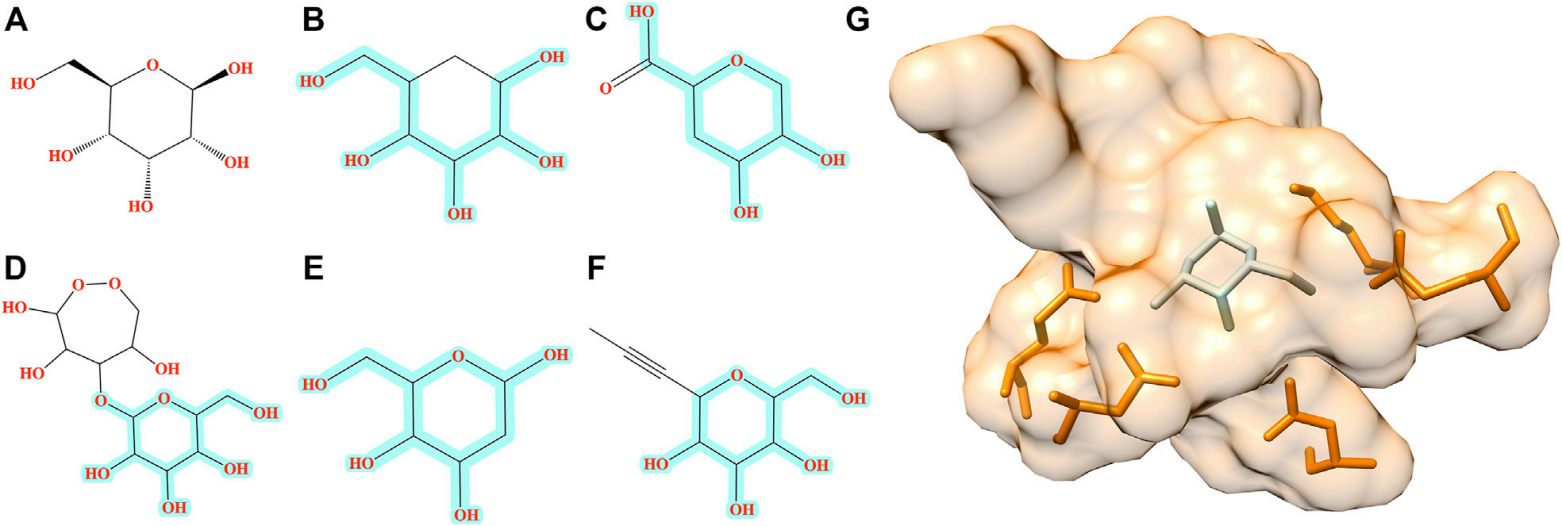
Examples of molecules generated by Pocket2Drug for a binding site in ALBP. **(A)** The label ligand, *ß*-d-allose. **(B–F)** Molecules constructed by Pocket2Drug with maximum common substructures to the label ligand highlighted in cyan. **(G)** Molecule shown in **E** (ice blue) docked to the binding site in ALBP (orange).

The improvement of Pocket2Drug over baseline methods is even more perceptible for ALBP where the distribution of TC similarities to the label ligand is shifted toward much higher values for molecules sampled from the Pocket2Drug model (Figure 4B). Differences between Pocket2Drug and both baseline methods are statistically significant with *p*-values close to 0. ALBP is a member of the ATP-binding cassette (ABC) transporter family facilitating the import and export of various molecules across the cell membrane (Fath and Kolter, 1993). ALBP binds *ß*-d-allose, shown in Figure 6A, with a *K*_d_ of 0.33 μm (Chaudhuri et al., 1999). In the crystal complex structure, *ß*-d-allose forms multiple interactions with the pocket residues of ALBP through the ring oxygen and five hydroxyl moieties (Chaudhuri et al., 1999). Selected compounds generated by Pocket2Drug are presented in Figures 6B–F. In addition to a substituted cyclohexane (Figure 6B), several substituted allose molecules (Figures 6C–F) sharing a high chemical similarity with the label ligand, *ß*-d-allose (Figure 6A), were constructed. Most of these molecules dock well to ALBP pocket with docking scores of 4.1, 3.7, 20.9, 3.5, and 9.8 for compounds shown in Figures 6B–F, respectively. Interestingly, a substituted cyclohexane in the molecule shown in Figure 6B adopts the chair conformation similarly to *ß*-d-allose bound to ALBP in the experimental complex structure. A compound shown in Figures 6E,G has the best docking score, whereas that shown in Figure 6D has less favorable docking score than those ligands having a comparable size to *ß*-d-allose because of the large substitution at 5′ position that does not fit in the binding pocket of ALBP. Docking results suggest that molecules generated by Pocket2Drug are capable of forming favorable interactions with the target pocket.

### Evaluation by Pocket Structure Alignments

**FIGURE 7:**
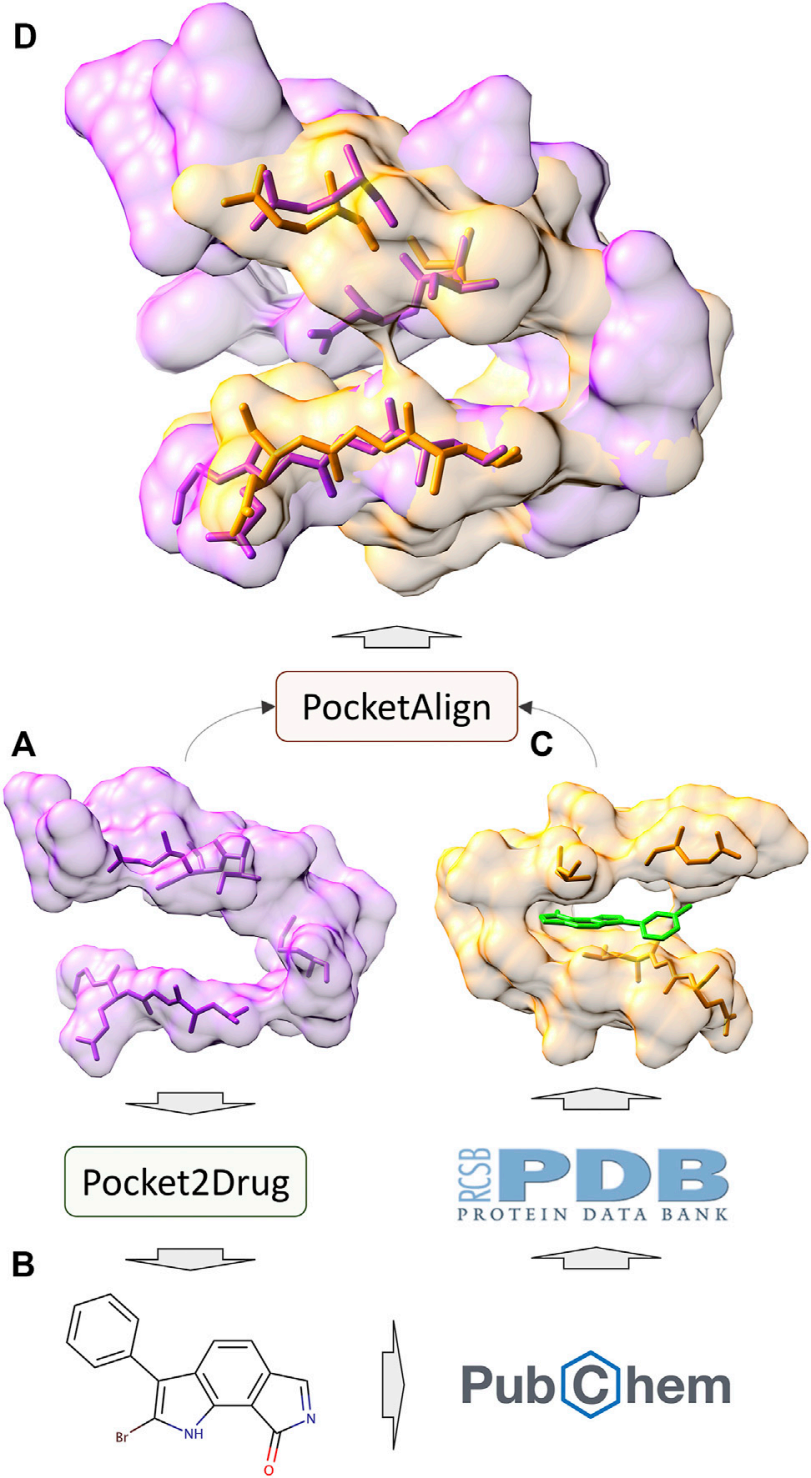
Flowchart of the evaluation by pocket structure alignments. For a target pocket **(A)**, a molecule is generated by Pocket2Drug **(B)**. This compound is then scanned through the PubChem BioAssay for similar molecules for which experimental complex structures are available in the Protein Data Bank. The extracted binding site **(C)** corresponding to the know interaction in PubChem BioAssay is structurally aligned to the target pocket by PocketAlign. A high-quality alignment **(D)** indicates that the generated molecule is likely to bind to the target pocket.

In addition to the assessment by ligand chemical similarity described above, the performance of Pocket2Drug is also evaluated with pocket structure alignments. This approach is based on an assumption that a molecule generated for the target pocket is a hit if a similar molecule binds to a site that is structurally similar to the target pocket (Govindaraj and Brylinski, 2018; Gaieb et al., 2019). A flowchart of the evaluation procedure is shown in Figure 7. For a target pocket in the testing set (Figure 7A), molecules generated by Pocket2Drug are ranked according to their frequencies and 100 of the most frequent molecules are selected. For each drug candidate (Figure 7B), chemically similar ligands with a TC of ≥0.7 are identified in the PubChem BioAssay dataset comprising 73,021 active interactions involving 919 unique proteins and 17,367 unique compounds (Wang et al., 2012). Next, the experimental complex structures of these ligands bound to similar proteins with a sequence identity of ≥70% to PubChem BioAssay targets are retrieved from the PDB. The extracted binding sites (Figure 7C) are finally structurally aligned to the initial target pocket with PocketAlign, an accurate method to superpose ligand binding sites in a sequence order-independent manner (Yeturu and Chandra, 2011). Essentially, this procedure validates molecules generated for target pockets by finding similar interactions that have already been determined experimentally through binding assays and protein crystallography.

**FIGURE 8:**
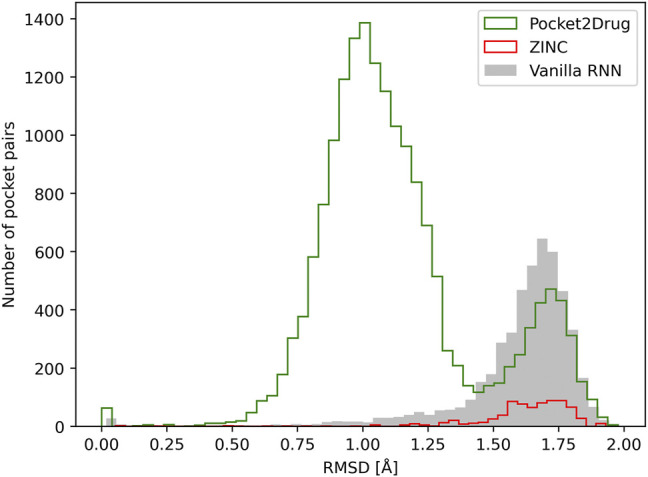
Assessment of the quality of pocket alignments constructed with PocketAlign. Alignment quality is evaluated by the root-mean-square deviation (RMSD) calculated over non-hydrogen atoms of binding residues. Target pockets are aligned to binding sites identified in the Protein Data Bank for molecules generated by Pocket2Drug (green) and two baselines, ZINC (red) and vanilla RNN (gray).

Similar to the evaluation protocol by ligand chemical similarity, Pocket2Drug is compared to ZINC and vanilla RNN. For each target pocket, 100 molecules from the ZINC database and 100 molecules generated by vanilla RNN are selected so that their molecular weight distributions match those calculated for compounds selected by Pocket2Drug. In terms of statistics, the number of pocket pairs used as input for structure alignments is 17,620 for Pocket2Drug, 6,307 for ZINC, and 6,694 for vanilla RNN. The number of valid pocket alignments constructed by PocketAlign (Yeturu and Chandra, 2011) are 16,987 (Pocket2Drug), 741 (ZINC), and 4,902 (vanilla RNN). A valid pocket alignment has the RMSD of ≤2 Å; higher RMSD values indicate that two pockets are structurally dissimilar. According to this criterion, as many as 96.4% of validation pairs of pockets identified using output molecules generated by Pocket2Drug produce valid structure alignments, while these percentages are notably lower for ZINC (11.7%) and vanilla RNN (73.2%). The distribution of the RMSD scores of pocket alignments for all tested methods is presented in Figure 8. Not only using molecules selected by Pocket2Drug results in the highest percentage of valid structure alignments, but also RMSD values for these superpositions are generally much lower compared to ZINC and vanilla RNN. The mean RMSD scores for pocket2Drug, ZINC, and vanilla RNN are 1.1 Å, 1.6 Å, and 1.6 Å, respectively.

**FIGURE 9:**
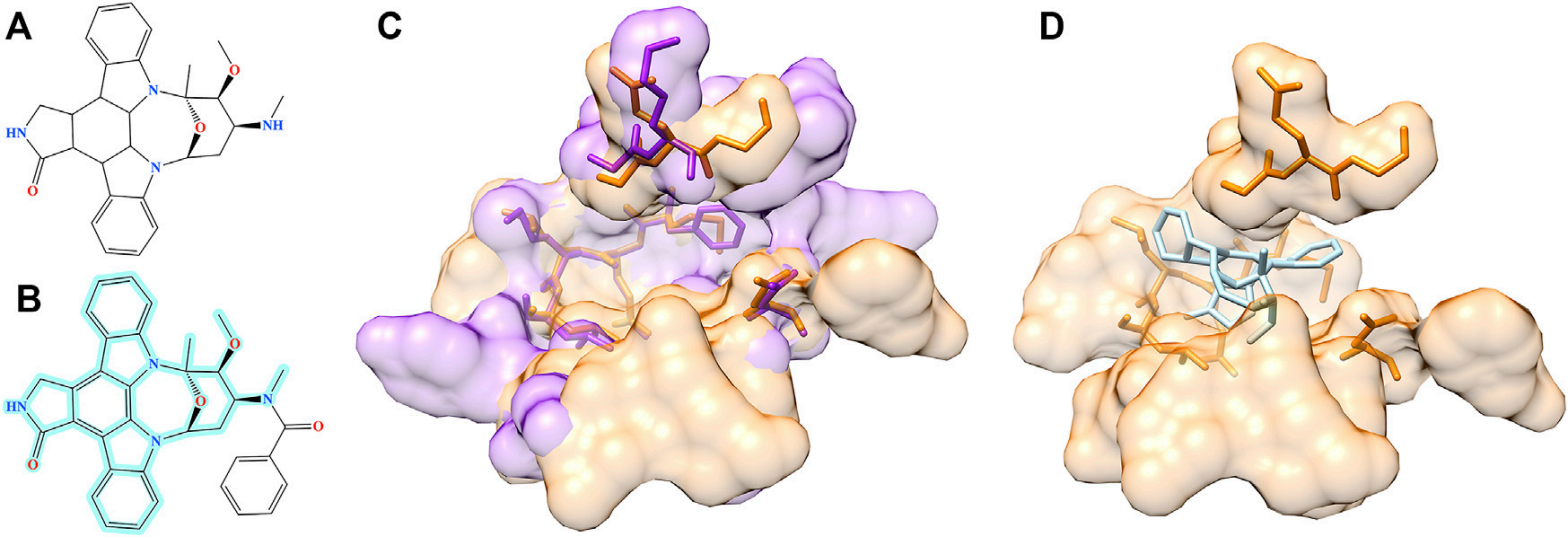
Example of the evaluation by pocket alignment for a binding site in MSK1. **(A)** A molecule generated by Pocket2Drug at rank 12. **(B)** A similar compound, midostaurin, with the maximum common substructure to Pocket2Drug molecule highlighted in cyan. **(C)** A structure alignment between the target binding site in MSK1 (orange) and midostaurin binding pocket in DYRK1A (purple). **(D)** The molecule generated by Pocket2Drug (ice blue) docked to the target site in MSK1 (orange) with fkcombu.

Structure alignment results demonstrate that for a large number of molecules generated by Pocket2Drug for target pockets, there are experimentally determined interactions between chemically similar ligands binding to structurally similar pockets. Two representative cases are selected to exemplify the evaluation by pocket structure alignments. The first target pocket is a nucleotide binding site in MSK1 used in the previous section to illustrate the results of the evaluation by ligand chemical similarity. Among molecules generated by Pocket2Drug, a compound ranked 12 with the frequency of 21 (Figure 9A) is chemically similar to midostaurin (PubChem-CID: 9829523, Figure 9B), a protein kinase C (PKC) inhibitor (Eder et al., 2004) used to treat systemic mastocytosis, acute myeloid leukemia, and mast cell leukemia (National Cancer Institute Dictionary, 2021). According to the bioassay data (PubChem-BAID: 208295368), midostaurin inhibits PKC-α isoform with the half-maximal inhibitory concentration (IC_50_) of 22 nm (Millward et al., 2006). Midostaurin has been co-crystalized with the human dual specificity tyrosine-phosphorylation-regulated kinase 1A (DYRK1A, 25% sequence identity with PKC-α) with the equilibrium dissociation constant (*K*_d_) of 100 nm (PDB-ID: 4nct) (Alexeeva et al., 2015). Figure 9C shows the structure alignment constructed by PocketAlign between AMP-PNP binding pocket in MSK1 and midostaurin binding pocket in DYRK1A. Despite a low global sequence identity between these proteins of only 26%, their binding pockets are structurally highly similar with the RMSD of 0.90 Å. The compound generated by Pocket2Drug docks to the AMP-PNP binding pocket in MSK1 with a score of 58.5 (Figure 9D).

**FIGURE 10:**
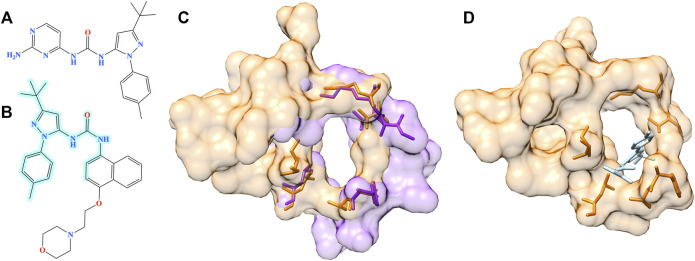
Example of the evaluation by pocket alignment for a binding site in Tie-2. **(A)** A molecule generated by Pocket2Drug at rank 9. **(B)** A similar compound, doramapimod, with the maximum common substructure to Pocket2Drug molecule highlighted in cyan. **(C)** A structure alignment between the target binding site in Tie-2 (orange) and doramapimod binding pocket in EphA2 (purple). **(D)** The molecule generated by Pocket2Drug (ice blue) docked to the target site in Tie-2 (orange) with fkcombu.

The second example is the human angiopoietin-1 receptor (Tie-2), an enzyme involved in vessel remodeling, branching, stability, and maturation (Yu, 2005). Using the binding site of Tie-2 as the input, Pocket2Drug generated a molecule shown in Figure 10A at rank 9 with a frequency of 5. This compound is chemically similar to doramapimod (PubChem-CID: 156422, Figure 10B), an inhibitor of ephrin type-A receptor 2 (EphA2) with a TC of 0.73. According to the bioassay data (PubChem-BAID: 40394839), doramapimod binds to EphA2 with a *K*_d_ of 0.37 nm and has been tested for its anti-proliferative activity in the SF-268 cell line. It inhibits the viability of EphA2 growth dependent glioblastoma cells with a half-maximal effective concentration (EC_50_) of 5 μm (Heinzlmeir et al., 2017). Despite a low global sequence identity of 37%, the structure alignment of binding sites in Tie-2 (PDB-ID: 2oo8) and EphA2 (PDB-ID: 5nkd) yields an RMSD of 0.95 Å (Figure 10C). Docking simulations with fkcombu confirmed that the molecule generated by Pocket2Drug fits well into the binding site of Tie-2 with a score of 24.3 (Figure 10D).

## Discussion

In this communication, we describe Pocket2Drug, a novel deep learning model employing an encoder-decoder architecture to predict binding molecules for a ligand binding site. Pocket2Drug was trained in an end-to-end supervised manner against a large collection of ligand-pocket pairs. The analysis of molecules generated by Pocket2Drug using two evaluation protocols based on ligand chemical similarity and pocket structure alignments revealed that this algorithm significantly improves the chances of finding binding ligands compared to traditional techniques. Pocket2Drug not only yields a high accuracy against ligand-free structures, but it also generalizes well to unseen data, *viz*. those pockets extracted from proteins that are different from training instances. These findings are particularly important in drug discovery against novel protein structures, where it can help significantly reduce the search space of drug candidates. In contrast to traditional virtual screening typically employing a library of 200,000 to over 1,000,000 molecules (Hughes et al., 2011), Pocket2Drug generates molecules that have high chances to bind to target pockets within a smaller sample of 81,920 compounds. Therefore, it can potentially decrease the number of molecules to be subjected to structure-based virtual screening from millions to tens of thousands.

Pocket2Drug can be improved by incorporating reinforcement learning imposing additional restraints on the synthetic accessibility, solubility, and toxicity of generated molecules, depending on a specific application. Additional improvements can also be achieved by applying a framework similar to the conditional recurrent neural network (cRNN), utilizing the RNN with the prior information (Xu et al., 2021), to the heterogeneous input data. In contrast to cRNN, in which the pre-computed information is used as the prior condition for RNN, Pocket2Drug is an end-to-end DNN, therefore the encoder is updated during training. Another difference is the data representation; cRNN uses a voxel representation as the prior information, whereas Pocket2Drug employs a computationally more efficient graph representation. Nonetheless, the heterogeneous pocket data can be combined by concatenating embedding vectors generated by different feature extractors in order to provide the prior information on ligand binding sites.

An attention mechanism was shown to significantly improve the performance of image captioning because it helps the model capture more semantically meaningful parts of images (Xu et al., 2015). We expect that the same methodology can be implemented in Pocket2Drug since pocket residues contribute differently to the formation of molecular interactions with binding ligands. These are examples of future research directions that will be explored to further improve the performance of Pocket2Drug in the discovery of novel biopharmaceuticals.

## Data Availability

The datasets presented in this study can be found in online repositories. The names of the repository/repositories and accession number(s) can be found below: https://github.com/shiwentao00/Pocket2Drug, https://osf.io/qacwj/.
